# Discovery of Chemical Constituents with Anti-Atopic Dermatitis Properties from *Aster koraiensis*

**DOI:** 10.3390/molecules29215002

**Published:** 2024-10-22

**Authors:** Ji-Young Kim, Hye-Min Kim, So-Ri Son, Hyo-Jin An, Dae Sik Jang

**Affiliations:** 1 Department of Biomedical and Pharmaceutical Sciences, Graduate School, Kyung Hee University, 26 Kyungheedae-ro, Dongdaemun-gu, Seoul 02447, Republic of Korea; k_christina@khu.ac.kr; 2 Department of Oriental Pharmaceutical Science, College of Pharmacy, Kyung Hee University, 26 Kyungheedae-ro, Dongdaemun-gu, Seoul 02447, Republic of Korea; mins7576@daum.net; 3 College of Pharmacy, Kyung Hee University, 26 Kyungheedae-ro, Dongdaemun-gu, Seoul 02447, Republic of Korea; allosori@khu.ac.kr

**Keywords:** *Aster koraiensis*, anti-atopic dermatitis, polyacethylenes, saponins, benzoic acid derivative

## Abstract

Atopic dermatitis is an inflammatory dermatological disease characterized by persistent scratching and recurrent eczema. Due to the influence of environmental variables on the cause of this disease, there remains an ongoing interest in the development of therapeutic interventions. Previous studies have shown that various plants of the genus *Aster* and its derived phytochemicals possess efficacy in treating inflammatory-mediated diseases, including atopic dermatitis. Therefore, the present study investigated a potential compound with anti-atopic dermatitis properties derived from *Aster koraiensis* leaves, specifically targeting HaCaT keratinocyte cells. First, we isolated eleven compounds with three unknown compounds, including two polyacetylenes (**1** and **3**) and a benzoic acid derivative (**4**). The chemical structures of the isolates were elucidated by 1D and 2D NMR, specific rotation, acid hydrolysis, and quantum chemical calculations. Next, we treated an *A*. *koraiensis* extract and all isolates to HaCaT keratinocyte, followed by stimulation with TNF-α/IFN-γ. Among bioactive compounds, astersaponin J (**7**) exhibited a significant reduction in the levels of inflammatory cytokines associated with atopic dermatitis at a concentration of 2.5 μM. These findings suggest that chemicals obtained from an *A*. *koraiensis* 95% ethanol extract and derived compounds are potential therapeutics to help reduce the immunological response driven by atopic dermatitis.

## 1. Introduction

Atopic dermatitis is a chronic, recurrent inflammatory dermatological disease usually occurring during childhood [1]. Its symptoms are characterized by persistent itching, dry skin, and distinctive dermatitis. There are two main causes of this disease: genetic factors and environmental factors, such as consumption of junk food or exposure to particulate matter [2,3]. Currently, the treatment of atopic dermatitis relies upon the utilization of topical corticosteroids. However, prolonged administration of these agents is associated with negative effects [4]. Improved pharmaceutical agents with reduced adverse effects have been formulated, but instances of adverse effects, including the development of skin malignancies, continue to be observed [5]. Hence, it is necessary to investigate alternative remedies using bioactive compounds that enhance the efficacy of treatment and minimize the adverse effects.

The genus *Aster* is a large group of plants widely distributed worldwide [6]. Various species of the *Aster* genus have been widely used for inflammation-related diseases in traditional medicines. Recently, previous studies have shown that various species within the *Aster* genus possess properties that inhibit skin inflammatory responses. For example, *A*. *glehni* extract has been shown to have anti-inflammatory effects in sodium dodecyl sulfate (SDS) or 2,4-dinitrochlorobenzene (DNCB)-induced HaCaT keratinocytes through the regulation of TRPV4, PPARδ, and the AMPK pathway [7]. Similarly, *A*. *yomena* has been reported to suppress proinflammatory cytokines by inhibiting phosphorylated Nrf2 and NF-κB p65 signals associated with inflammatory damage in UVB-irradiated HaCaT cells [8]. However, given that the *Aster* genus comprises approximately 200 species and each produces various secondary metabolites, further research is needed to explore additional *Aster* species and their bioactive compounds with potential anti-inflammatory effects on the skin.

*Aster koraiensis* Nakai (synonym: *Gymnaster koraiensis*; family: Compositae), an endemic plant of Korea, has been widely used in traditional Korean medicine to treat inflammation-mediated diseases, such as chronic bronchitis or pneumonia. Phytochemical investigations have revealed that polyacetylenes, sesquiterpenes, triterpenoids, flavonoids, and caffeoylquinic acid are principal secondary metabolites in *A*. *koraiensis* [9,10,11,12,13,14]. In addition, several investigations of the pharmacological uses for *A*. *koraiensis* reported to possess properties associated with antioxidative, anti-inflammatory, anticancer, and hepatoprotective activities [9,10,15,16]. Nevertheless, there is a lack of studies for the immune-inflammatory response in atopic dermatitis. Moreover, the bioactive compounds from *A*. *koraiensis* in atopic dermatitis have not been conducted. Therefore, the present study investigated the effects of *A*. *koraiensis* leaf extract and its compounds on TNF-α/IFN-γ-induced HaCaT keratinocytes to identify potential therapeutic agents for atopic dermatitis.

## 2. Results and Discussion

### 2.1. Isolation and Structure Elucidation of Compounds from A. koraiensis

**Figure 1:**
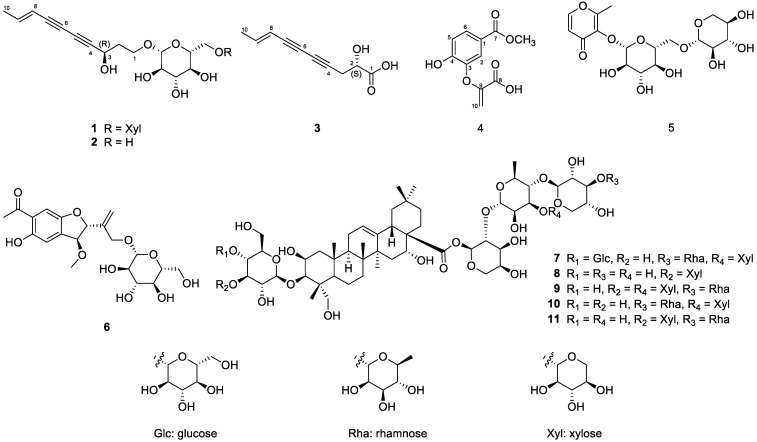
Chemical structures of the isolated compounds **1**–**11** from the leaves of *A. koraiensis*.

Our previous study demonstrated saponins, sesquiterpene glycosides, polyacetylenes, and dicaffeoylquinic acid as principal compounds from a 95% EtOH extract of *A*. *koraiensis* leaves [11,12]. As part of our ongoing research to identify novel bioactive compounds with anti-atopic dermatitis, a total of 11 compounds were identified through a spectroscopic data analysis and a literature review (Figure 1). Among them, three compounds (**1**, **3**, and **4**) were unreported, and their chemical structures were elucidated accordingly.

**Figure 2:**
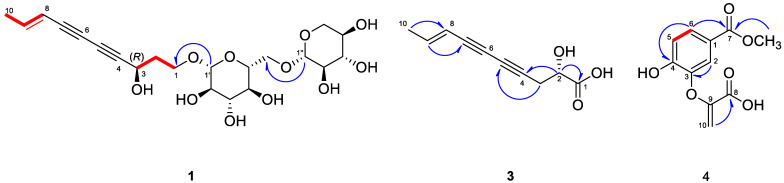
Key COSY (red line) and HMBC (blue arrow) correlations of compounds **1**, **3**, and **4**.

Compound **1** was isolated as a yellow powder, and its molecular formula was established as C_21_H_30_O_11_ by HR-ESI-MS with a molecular ion peak at *m*/*z* 459.1872 [M+H]^+^ (calcd for C_21_H_31_O_11_, 459.1862; Figure S1). The UV spectrum exhibited characteristic ene-diyne chromophore absorptions at 236, 254, and 266 nm. The ^1^H NMR spectrum exhibited a methyl proton at *δ*_H_ 1.80 (3H, dd, *J* = 7.0, 2.0 Hz, H-10), an oxygenated methine proton at *δ*_H_ 4.65 (1H, t, *J* = 7.0 Hz, H-3), two anomeric protons at *δ*_H_ 4.31 (1H, d, *J* = 7.5 Hz, H-1″) and 4.26 (1H, d, *J* = 8.0 Hz, H-1′), and two olefinic protons at *δ*_H_ 6.33 (1H, dd, *J* = 7.0, 2.0 Hz, H-9) and 5.59 (1H, dt, *J* = 16.0, 2.0 Hz, H-8) (Figure S2 and Table 1). By analyzing ^13^C and HSQC NMR spectroscopic data, 21 carbon signals were identified, including 1 methyl at *δ*_C_ 19.1, 2 olefinic carbons at *δ*_C_ 145.3 and 110.7, 4 quaternary carbons at *δ*_C_ 83.9, 78.3, 72.6, and 69.9, and 2 anomeric carbons at *δ*_C_ 105.7 and 104.8 (Figure S2 and Table 1). In the ^1^H-^1^H COSY spectrum, sequential correlations from H-1 to H-3 and from H-8 to H-10 were observed, suggesting the structural connectivity within the polyacetylene moiety, as shown in Figure 2. Compound **1** was similar to gymnasterkoreaside A (**2**), except for one more sugar moiety [17]. The positions of sugar moieties were determined using HMBC correlation from H-1′ to C-1 and from H-1″ to C-6′ (Figure 2 and Figure S6).

To determine the absolute configuration, an acid hydrolysis for **1** was conducted and led to producing the sugar moiety of **1** and the aglycone moiety of **1**—known as gymnasterkoreayne A [17]. The sugars were confirmed as d-xylose and d-glucose with HPLC by comparing analyses with standard samples (Figures S18 and S19). The specific rotation for aglycone of **1** (−11.6°) was similar to those for gymnasterkoreayne A (−14°) compared to the literature data, indicating that they have the same configuration [17]. Therefore, the structure of **1** was elucidated as (3*R*)-8-decene-4,6-diyne-1,3-diol-1-*O*-β-d-xylopyranosyl-(1⟶6)-*O*-β-d-glucopyranoside, named as gymnasterkoreaside C.

**Figure 3:**
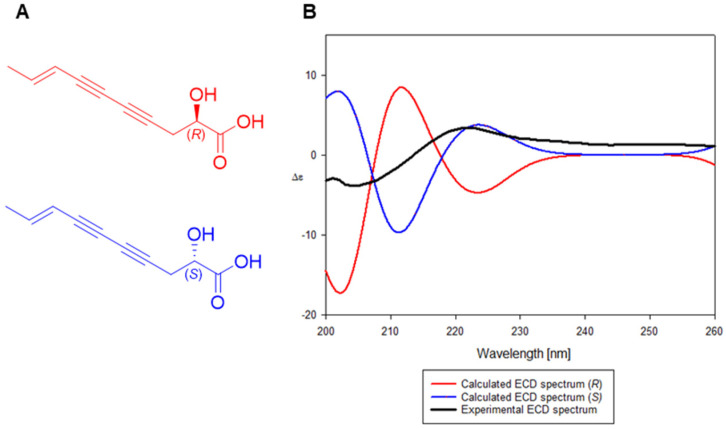
(**A**) Plausible absolute configurations of compound **3**. (**B**) Measured and calculated electronic circular dichroism (ECD) spectrum of compound **3**.

Compound **3** was isolated as a yellow oil, and its molecular formula was determined by HR-ESI-MS with a molecular ion peak at *m*/*z* 179.0708 [M + H]^+^ (calcd for C_10_H_11_O_3_, 179.0704; Figure S8). The UV spectrum exhibited an absorption band at 254, 265, and 282 nm. The ^1^H NMR spectrum of **3** exhibited a methyl signal at *δ*_H_ 1.78 (3H, H-10), a methylene signal at *δ*_H_ 2.52 (2H, H-3), two olefinic signals at *δ*_H_ 5.55 (1H, H-8) and *δ*_H_ 6.29 (1H, H-9), and an oxygenated methine signal at *δ*_H_ 4.73 (1H, H-2) (Figure S9 and Table 1). The ^13^C NMR spectrum showed ten signals including quaternary carbons at *δ*_C_ 69.6, 72.7, 78.2, and 83.6 (Figure S10 and Table 1). The planar structure of **3** was further determined using an HMBC experiment by the following correlations: H-2 to C-1/4, H-3 to C-5, H-9 to C-7, and H-10 to C-8 (Figure 2 and Figure S11). To determine the absolute configuration of **3**, we performed an ECD experiment and observed the positive cotton effect at 225 nm (Figure 3). Therefore, the structure of **3** was elucidated as (2*S*,8*E*)-2-hydroxydeca-8-en-4,6-diynoic acid.

Compound **4** was isolated as a white powder, in which the molecular formula was established as C_11_H_10_O_6_ by HR-ESI-MS (*m*/*z* = 239.0559 [M + H]^+^; calcd for C_11_H_11_O_6_, 239.0550; Figure S12) with a melting point of 195.6 °C. The ^1^H-NMR spectrum of compound **5** appeared as ABX system signals at *δ*_H_ 7.81 (1H, dd, *J* = 8.5, 1.5 Hz, H-6), 7.72 (1H, d, *J* = 1.5 Hz, H-2), and 7.68 (1H, d, *J* = 8.5 Hz, H-5), with a sharp singlet methoxy signal observed at *δ*_H_ 3.88 (3H, s, 7-OCH_3_), and one exomethylene signal appeared at *δ*_H_ 5.44 (d, *J* = 1.5 Hz, H-10a) and 4.43 (d, *J* = 2.5 Hz, H-10b) (Figure S20 and Table 2). The ^13^C-NMR spectrum showed the presence of 11 carbons, indicating one methoxy signal (*δ*_C_ 52.7), three olefinic carbons in aromatic rings (*δ*_C_ 127.2, 124.0, 124.2), one exomethylene carbon (*δ*_C_ 98.6), and six quaternary carbons (*δ*_C_ 169.2, 167.5, 157.4, 149.4, 148.4, 128.4) (Figure S21 and Table 2). Following these data, compound **4** was predicted to be a phenolic compound. These data were very similar to dehydrochorismic acid, except for the methoxycarbonyl location [18]. The position of the methoxy signal was confirmed by an HMBC spectrum, indicating a long-range correlation from 7-OCH_3_ to C-7 and from H-6 to C-7, suggesting the structure of compound **4** as a dehydrochorismic acid methyl ester.

The structures of eight known compounds identified as gymnasterkoreaside A (**2**; C_16_H_22_O_7_; ESI-LR-MS: *m*/*z* 327.1 [M+H]^+^) [17], spatholosineside A (**5**; C_17_H_24_O_12_; *m*/*z* 421.1 [M+H]^+^) [19], (2*R*,3*S*)-6-acetyl-2-[1-*O*-(-β-d-glucopyranosyl)-2-propenyl]-5-hydroxy-3-methoxy-2,3-dihydrobenzofuran (**6**; C_20_H_26_O_10_; *m*/*z* 427.1 [M+H]^+^) [20], astersaponin J (**7**; C_69_H_112_O_36_; *m*/*z* 1517.7 [M+H]^+^), astersaponin L (**8**; C_57_H_92_O_27_; *m*/*z* 1209.6 [M+H]^+^), astersaponin I (**9**; C_68_H_110_O_35_; *m*/*z* 1487.6 [M+H]^+^) [11], 3-*O*-β-d-glucopyranosyl-2*β*,3*β*,16*α*,23-tetrahydroxyolean-12-en-28-oic acid 28-*O*-α-l-rhamnopyranosyl-(1⟶3)-β-d-xylopyranosyl-(1⟶4)-[β-d-xylopyranosyl-(1⟶3)]-α-l-rhamnopyranosyl-(1⟶2)-α-l-arabinopyranoside (**10**; C_63_H_102_O_31_; *m*/*z* 1354.6 [M+H]^+^) [21], and conyzasaponin J (**11**; C_63_H_102_O_31_; *m*/*z* 1354.6 [M+H]^+^) [22] were verified by comparison with the previously reported literature. Through this study, compound **5** is reported for the first time as constituents of *A. koraiensis*.

### 2.2. Anti-Atopic Dermatitis Effects of 95% EtOH Extract of A. koraiensis Leaf Extract and Isolates on HaCaT Keratinocytes

During the development of atopic dermatitis, keratinocytes enhance the inflammatory response due to the increased levels of pro-inflammatory cytokines, such as TNF-α or IFN-γ [23]. Sequentially, stimulated keratinocytes produce and release inflammatory mediators, including IL-1β and IL-6, and even produce additional TNF-α and IFN-γ [23]. Also, recent scientific shreds of evidence demonstrate that the levels of these inflammatory mediators tend to be excessively elevated in atopic dermatitis patients [24,25]. Thus, we evaluated whether the 95% EtOH extract of *A*. *koraiensis* leaves and its isolated compounds could regulate inflammatory mediators in TNF-α/IFN-γ-stimulated HaCaT keratinocytes.

**Figure 4:**
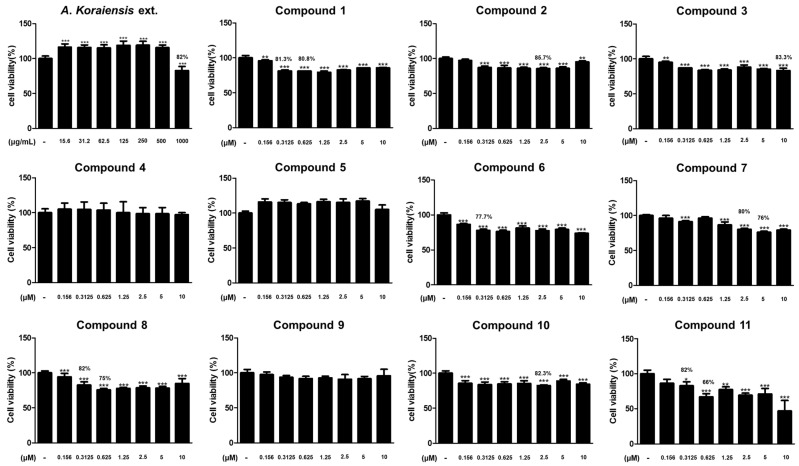
Effects of *A. koraiensis* extract and isolated compounds **1**–**11** on cell viability in HaCaT keratinocytes. HaCaT keratinocytes were incubated with various concentrations of *A. koraiensis* extract or isolated compounds **1**–**11** for 24 h, and cell viability was measured by MTT assay. * *p* < 0.05, ** *p* < 0.01, and *** *p* < 0.001 vs. *A. koraiensis* non-treated group.

First, the MTT assay was conducted to confirm cell viability against HaCaT keratinocytes after treatment of the 95% EtOH extract of *A*. *koraiensis* leaves and its isolated compounds. To identify the optimal concentration range for further cytokine studies, the concentration ranges of the samples were set to 15.6–1000 μg/mL for the extract and 0.156–10 μM for each compound. As shown in Figure 4, the 95% EtOH extract of *A*. *koraiensis* leaves did not affect cell viability at concentrations of 15.6–500 μg/mL, and a significant decrease in cell viability was observed at the highest concentration of 1000 μg/mL (82.8 ± 5.8%, *p* < 0.001). Compounds **6**, **8**, and **11** showed dose-dependent decreases in cell viability, with viability rates under 80% at most concentrations. Hence, these compounds were excluded from subsequent experiments on the level of inflammatory mediators because of their potential cytotoxicity. Compound **1** showed over 80% viability, but, due to the limited quantity, further studies could not be conducted. In contrast, compounds **2**–**5**, **7**, **9**, and **10** maintained cell viability rates above 80% across the tested concentration range, indicating that they have no apparent cytotoxicity on HaCaT cells. Based on the MTT results, these compounds were selected to assess the expression level of inflammatory mediators after the stimulation of TNF-α/IFN-γ.

Next, IL-1β and IL-6 production were evaluated as they are key inflammatory mediators involved in the pathogenesis of atopic dermatitis. IL-1β and IL-6 drive the inflammatory cascade in atopic dermatitis, contributing to the activation of inflammatory T cells and subsequent skin barrier dysfunction [26]. Thus, targeting IL-1β and IL-6 could be a promising approach in the treatment of atopic dermatitis.

**Figure 5:**
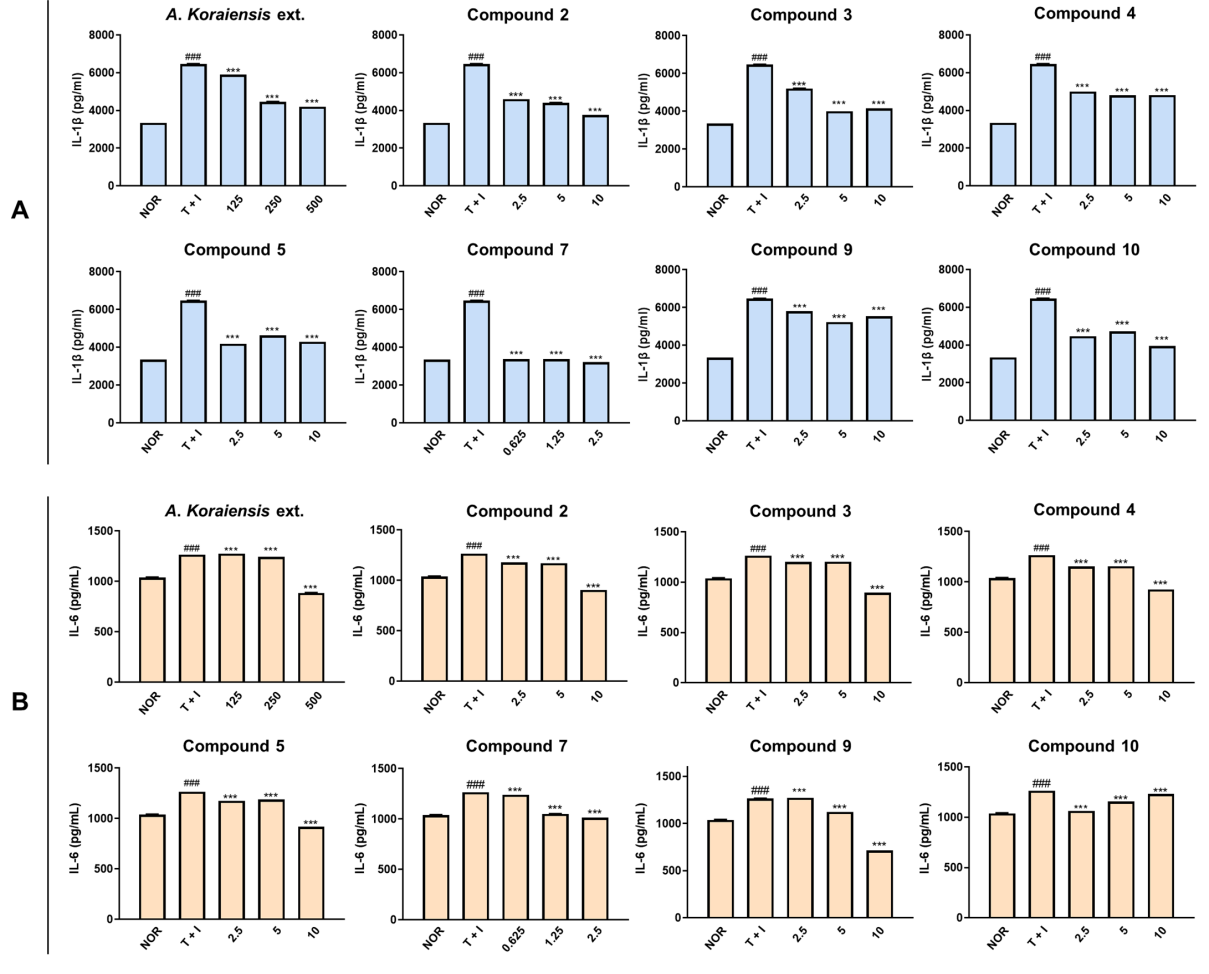
Effects of *A*. *koraiensis* extract and isolates on the atopic dermatitis-related cytokines in TNF-α/IFN-γ-stimulated HaCaT keratinocytes. HaCaT keratinocytes were pre-treated with *A*. *koraiensis* extract and its isolates for 1 h and then stimulated with TNF-α/IFN-γ (10 ng/mL) for 24 h. The level of IL-1β (**A**) and IL-6 (**B**) in cell culture supernatants was measured using each specific ELISA kit. All data shown represent the mean ± standard deviation (SD) of triplicate independent experiments. ^###^ *p* < 0.001 vs. the control group; *** *p* < 0.001 vs. TNF-α/IFN-γ-treated group.

As shown in Figure 5A, IL-1β was significantly increased by TNF-α/IFN-γ stimulation (T+I, *p* < 0.001) compared to the non-stimulated control (NOR). In the pretreatment group of the *A*. *koraiensis* extract, IL-1β production induced by TNF-α/IFN-γ was suppressed in a dose-dependent manner (125–500 μg/mL). Additionally, all groups treated with selected compounds exhibited inhibitory effects on IL-1β levels against TNF-α/IFN-γ-stimulated HaCaT cells at non-cytotoxic concentrations. Among isolates, astersaponin J (**7**) reduced IL-1β levels to those of NOR, even at the lowest concentration (0.625 μM, *p* < 0.001). Additionally, the polyacetylenes **2** and **3** also demonstrated significant IL-1β suppression (*p* < 0.001), reducing IL-1β levels to those observed in the NOR group.

Meanwhile, IL-6 expression was amplified in the T+I group (*p* < 0.001), similar to the increase pattern observed with IL-1β (Figure 5B). However, *A*. *koraiensis* extract displayed a significant inhibitory effect only at a concentration of 500 μg/mL. The pretreatment groups of selected compounds dose-dependently diminished IL-6 levels on TNF-α/IFN-γ-induced HaCaT cells, except for compound **9**, which showed a slight increasing trend at higher concentrations.

**Figure 6:**
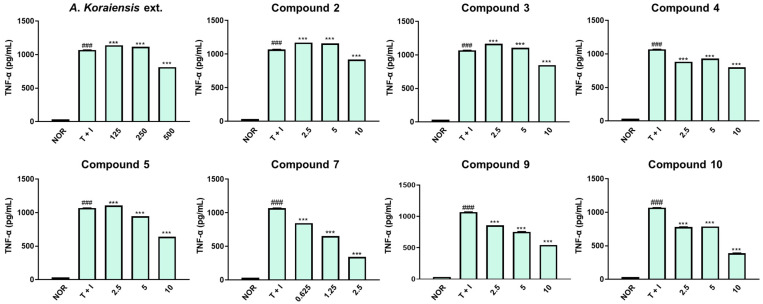
Effects of *A*. *koraiensis* extract and isolated compounds on TNF-α secretion in TNF-α/IFN-γ-stimulated HaCaT keratinocytes. HaCaT keratinocytes were pre-treated with *A*. *koraiensis* extract and its isolates for 1 h and then stimulated with TNF-α/IFN-γ (10 ng/mL) for 24 h. The level of TNF-α in cell culture supernatants was measured using an ELISA kit. All data shown represent the mean ± standard deviation (SD) of triplicate independent experiments. ^###^ *p* < 0.001 vs. the control group; *** *p* < 0.001 vs. TNF-α/IFN-γ-treated group.

Lastly, we assessed the TNF-α secretion induced by TNF-α/IFN-γ exposure. TNF-α is reproduced in stimulated keratinocytes, contributing to the progression of atopic dermatitis into chronic inflammation. TNF-α plays a crucial role in the early stages of the inflammatory response and induces the expression of successive inflammatory cytokines such as IL-1β and IL-6 [27,28]. Figure 6 presents that TNF-α release into the supernatant increased in TNF-α/IFN-γ (*p* < 0.001). In line with the IL-6 results, the *A*. *koraiensis* extract inhibited TNF-α production at a concentration of 500 μg/mL and exhibited a dose-dependent inhibition of TNF-α production (*p* < 0.001). The pretreatment groups of isolated compounds demonstrated inhibitory properties at 10 μM (*p* < 0.001), indicating that treatment with individual compounds effectively suppressed TNF-α levels compared to treatment with the *A*. *koraiensis* extract. Specifically, saponins (**7**, **9**, and **10**) from *A*. *koraiensis* exhibited a dose-dependent inhibitory effect in comparison to other compounds. Astersaponin I (**7**) shows the strongest inhibitory effect at a concentration of 2.5 μM among the compounds.

Based on the above results, *A*. *koraiensis* and its derived compounds have been identified as drug candidates for atopic dermatitis. To the best of our knowledge, the present study first evaluated the anti-atopic properties of aliphatic C_10_ polyacetylenes (**2** and **3**). These compounds effectively inhibited the production of pro-inflammatory cytokines in TNF-α/IFN-γ-simulated HaCaT keratinocytes at 10 μM. To validate and compare their effects, a structure-activity relationship of these compounds focusing on the presence of sugars and substituent variations must be investigated.

Previous studies have reported the anti-inflammatory properties of benzoic acid derivatives, maltol derivatives, and saponins in the treatment of atopic dermatitis. For example, methyl vanillate, a benzoic acid derivative, attenuates the mitogen-activated protein kinase (MAPK) pathway, which triggers the secretion of pro-inflammatory mediators in keratinocytes stimulated by TNF-α/IFN-γ [29]. Maltol reduces the atopic dermatitis-like symptoms in a 2,4-dinitrochlorobenzene-induced atopic mouse model [30]. Platycodin D, a saponin isolated from *Platycodon grandiflorum*, alleviates atopic dermatitis-like skin lesions through the regulation of NF-κB, STAT1, and heme oxygenase-1 [31]. However, this is the first report on the anti-inflammation properties of compounds **4**, **5**, **7**, **9**, and **10** against TNF-α/IFN-γ-induced HaCaT cells. Furthermore, astersaponin I (**7**) consistently suppressed multiple pro-inflammatory cytokines (IL-1β, IL-6, and TNF-α) even at the lowest concentration (0.625 μM), highlighting it as a potential therapeutic candidate for atopic dermatitis. However, further mechanistic studies and in vivo experiments are needed to validate the effect of astersaponin I (**7**) and to support its potential application in clinical trials.

## 3. Materials and Methods

### 3.1. General Experimental Procedures

On an Optizen pop (Mecasys, Daejeon, Republic of Korea), UV spectra were evaluated. Melting points in open capillary tubes were measured using MPA 100 (Stanford Research Systems, Sunnyvale, CA, USA). Using a 10 cm microcell, optical rotations were measured on a Jasco P-2000 polarimeter (JASCO, Tokyo, Japan). JNM-ECZ500R (JEOL, Tokyo, Japan) 500 MHz NMR spectra were obtained. A Q-TOF micromass spectrometer (Waters, Milford, MA, USA) was used to obtain the HR-Mass spectra. Silica gel 60 F254 (Merck, Kenilworth, MA, USA) and RP-18 F254S (Merck) plates were used for TLC studies. Plates were dipped into 20% (*v*/*v*) H_2_SO_4_ reagent (Samchun) and heated to 110 °C for five to ten minutes in order to visualize the compounds. Column chromatography was performed using Sephadex LH-20 (Amersham Pharmacia Biotech, Buckinghamshire, United Kingdom), reversed-phase silica gel (YMC Co., Ltd., Kyoto, Japan; ODS-A 12 nm S-150 μm), Diaion HP-20 (Mitsubishi, Tokyo, Japan), and Merck 60A, 230–400 mesh ASTM silica gel. For flash chromatography, prepackaged cartridges containing Redi Sep-Silica (12 g, 24 g, 40 g, Teledyne Isco, Lincoln, NE, USA) and Redi Sep-C18 (13 g, 26 g, 43 g, 130 g, Teledyne Isco) were utilized. The flash purification system (Combi Flash Rf, Teledyne Isco) was used for flash chromatography. HPLC was carried out using a Gemini NX-C18 110A column (250 × 21.2 mm i.d. 5 μm, Phenomenex, Torrance, CA, USA) and a Waters purification system (1525 pump, PDA 1996 detector). For this investigation, all solvents were distilled prior to chromatographic separations.

### 3.2. Plant Material

*Aster koraiensis* Nakai (Compositae) leaves were gathered in 2017 in Pyeongchang, Gangwon-Do, Republic of Korea. The origin of the plant was authenticated by one of the authors (D.S.J.), and a voucher specimen (ASKO1-2017) was deposited at the College of Pharmacy, Kyung Hee University, Republic of Korea.

### 3.3. Extraction and Isolation

The extraction procedure was followed by a previously described method [11]. Then, the 95% EtOH extract of *A*. *koraiensis* leaves (500 g) was chromatographed over Diaion HP-20 (9.8 × 63.0 cm) eluting with an acetone-H_2_O gradient (from 0:1 to 1:0 *v*/*v*) to afford 28 fractions (C1~C28).

Fraction C4 (11.82 g) was separated into five subfractions by using Sephadex LH-20 (4.8 × 63.0 cm) with 50% acetone (C4-1~C4-5). Subfraction C4-2 (2.99 g) was subjected to use Sephadex LH-20 (3.6 × 63.0 cm) with a MeOH-H_2_O mixture (1:1 *v*/*v*), yielding five subfractions (C4-2-1~C4-2-5). Subfraction C4-2-2 (1.64 g) was chromatographed over a silica gel CC (230-400 mesh; 3.6 × 27.5 cm) as a stationary phase with CH_2_Cl_2_-MeOH-H_2_O [80:18:2 *v*/*v*/*v*; final stage, MeOH 100%] as a mobile phase to afford seven fractions (C4-2-2-1~C4-2-2-7). Compounds **4** (7.0 mg) and **5** (18.0 mg) were obtained by a flash chromatography system using a Redi Sep-C18 cartridge (130 g, MeOH-H_2_O, from 0:100 to 30:70 *v*/*v*) from C4-2-2-3 (397.2 mg).

Fraction C9 (3.64 g) was separated with Sephadex LH-20 CC (3.6 × 63.0 cm) with MeOH-H_2_O (1:1 *v*/*v*) to afford nine subfractions (C9-1~C9-9). Compound **1** (1.8 mg) was purified with a flash chromatography system with a Redi Sep-C18 cartridge (43 g, MeOH-H_2_O, 40:60~70:30, *v*/*v*) from subfraction C9-5.

Fraction C11 (10.0 g) was separated into seven subfractions (C11-1~C11-7) by Sephadex LH-20 CC (3.6 × 65.0 cm) with 50% acetone. Subfraction C11-1 (1.06 g) was fractionated by silica gel CC and afforded to three subfractions (C11-1-1~C11-1-3). The compounds **7** (32.0 mg), **8** (6.8 mg), **9** (99.6 mg), **10** (10.8 mg), and **11** (15.1 mg) were obtained by repeated Sephadex LH-20 CC and preparative HPLC from subfraction C11-1-1. Subfraction C11-3 (1.03 g) was chromatographed over a reversed-phase silica gel (3.7 × 28.0 cm) as a stationary phase with acetone-H_2_O gradient (from 30:70 to 60:40 *v*/*v*; final stage, MeOH 100%) as a mobile phase to generate 11 subfractions (C11-3-1~C11-3-11). Subfraction C11-3-5 (338.5 mg) was fractionated further with a flash chromatographic system with a Redi Sep-C18 cartridge (43 g, MeOH-H_2_O, 35:65 to 50:50 *v*/*v*) to afford compounds **2** (29.7 mg) and **6** (3.0 mg).

Fraction C17 (3.12 g) was separated into five subfractions (C17-1~C17-5) by using Sephadex LH-20 CC (4.0 × 69.0 cm) with MeOH-H_2_O (1:1 *v*/*v*; final stage, MeOH 100%). Subfraction C17-3 (300.0 mg) was fractionated with a flash chromatographic system with a silica cartridge (48 g, CH_2_Cl_2_-MeOH-H_2_O, 35:65 to 50:50, *v*/*v*) to afford compound **3** (1.5 mg).

#### 3.3.1. Gymnasterkoreaside C (**1**)

Pale yellowish powder; HR-ESI-MS (positive mode) *m*/*z* = 459.1872 [M + H]^+^ (calcd for C_21_H_31_O_11_, 459.1862); [α]_D_^25^: 148.0° (*c* 0.1, MeOH); UV (MeOH) λ_max_ 236 (1.36), 254 (3.84), 266 (4.31) nm; ^1^H and ^13^C NMR data in Table 1.

#### 3.3.2. (2*S*,8*E*)-Hydroxydeca-8-en-4,6-diynoic Acid (**3**)

Yellowish powder; HR-ESI-MS (positive mode) *m*/*z* = 179.0708 [M + H]^+^ (calcd for C_10_H_11_O_3_, 179.0704); UV (MeOH) λ_max_ 254 (4.81), 265 (5.02), 282 (5.10) nm; ^1^H and ^13^C NMR data in Table 1.

#### 3.3.3. Dehydrochorismic Acid Methyl Ester (**4**)

Pale yellowish powder; HR-ESI-MS (positive mode) *m*/*z* = 239.0559 [M + H]^+^ (calcd for C_11_H_11_O_6_, 239.0550); m.p. 195.6 °C; UV (ACN) λ_max_ 209, 211, 246, 286, 321, 374 nm; ^1^H and ^13^C NMR data in Table 2.

### 3.4. Acid Hydrolysis and Sugar Analysis

For the determination of absolute configuration, compound **1** (1.0 mg) was hydrolyzed with 2N HCl for an hour at 18 °C. The reaction was stopped with sodium bicarbonate and subsequently suspended with H_2_O-EtOAc (1:1, *v*/*v*). The EtOAc-soluble fraction of **1** was evaporated and dissolved with MeOH for measuring specific rotation. The water-soluble fraction was evaporated under N_2_ at 45 °C. Next, a derivatization of sugar was conducted for each sample to identify the absolute configuration. The resulting concentrate was dissolved with pyridine (500 μL) and l-cysteine methyl ester hydrochloride (1.2 mg) mixture and heated for an hour at 60 °C. Then, σ-tolyl isothiocyanate (20.0 μL) was added to the dissolved concentrate. Finally, the derivatized mixture (10.0 μL) was injected to HPLC with an isocratic system (A: 0.5% acetic acid in H_2_O, B: 0.5% acetic acid in ACN; 20%B, 0–40 min), and its chromatogram was compared to those of authentic standards using a PDA detector.

### 3.5. Computational Analysis

The 3D models of compounds were built using Chem3D modeling. A conformational structure analysis was performed by the Merck Molecular Force Field (MMFF) as implemented in the Spartan′18 software (Wavefunction, Inc., Irvine, CA, USA). Geometry optimization of the conformers was performed at the density functional theory (DFT) B3LYP/6–31+G (d,p) level by the Gaussian 16W software (Revision E.01; Gaussian, Inc., Wallingford, CT, USA; 2009). The time-dependent density functional theory (TDDFT) ECD calculations were performed at the CAM–B3LYP level with a polarizable continuum model calculation (CPCM) solvent model (acetonitrile) and at the DFT [B3LYP/6–31+G (d,p) basis set] level by using the Gaussian 16W software, respectively.

### 3.6. Cell Culture and Sample Treatment

HaCaT keratinocytes were generously provided by Professor Kyung-Tae Lee from Kyung Hee University. These cells were cultivated in Dulbecco’s Modified Eagle Medium (DMEM) supplemented with 10% fetal bovine serum (FBS), penicillin (100 U/mL), and streptomycin (100 μg/mL). They were maintained in a controlled incubation environment at 37 °C with 5% CO_2_. Cells were usually subcultured 2–3 times per week by seeding 4.0 × 10^5^ cells in a 100 mm culture dish using 1× Trypsin-EDTA solution. Subsequently, HaCaT keratinocytes were seeded at a density of 1 × 10^5^ cells per well for sample treatment and incubated for 24 h at 37 °C in a humidified atmosphere containing 5% CO_2_. Following this incubation period, the cells were subjected to treatment with each *A. koraiensis* extract and compounds at various concentrations for the duration of an hour before being stimulated with 10 ng/mL of TNF-α/IFN-γ for the specified period of time.

### 3.7. MTT Assay

Cell viability was assessed through a colorimetric MTT assay. HaCaT keratinocytes were seeded at a density of 5 × 10^4^ cells per well in 96-well plates and allowed to adhere for 24 h. Subsequently, the cells were subjected to treatment with a medium containing varying concentrations of *A. koraiensis* extract and compounds and incubated for 24 h. On the following day, 50 μL of MTT (5 mg/mL) was administered to the cells and incubated for 4 h. The formazan precipitate was dissolved in dimethyl sulfoxide (DMSO), and the absorbance was measured at 540 nm using an Epoch microplate spectrometer (BioTek, Winooski, VT, USA).

### 3.8. Enzyme-Linked Immunosorbent Assay (ELISA) for Cytokine Analysis

Cell culture supernatants were collected approximately 24 h after treatment with each *A. koraiensis* extract and compounds and subsequently stored at a temperature of −70 °C. In accordance with the manufacturer’s instructions, the expression levels of IL-1β, TNF-α, and IL-6 were evaluated using ELISA kits (Bio-Rad Laboratories, Inc., Hercules, CA, USA).

### 3.9. Statistical Analysis

Statistical analyses were performed using GraphPad Prism version 5 (GraphPad Software Inc., La Jolla, CA, USA). Data are presented as mean ± standard deviation (SD). Data were analyzed using a one-way analysis of variance (ANOVA), and statistical significance was defined using Dunnett’s multiple comparison test at *p* < 0.05.

## 4. Conclusions

In this study, we investigated the anti-atopic effects of ethanolic extracts from the leaves of *A. koraiensis* and isolated 11 compounds, including three previously undescribed compounds (**1**, **3**, and **4**) via repetitive chromatography. Four unknown compounds were identified as polyacetylenes (**1** and **3**) and a benzoic acid derivative (**4**) by a comprehensive analysis of spectroscopic data, hydrolysis, and quantum chemical calculations. The known compounds were chemically elucidated as polyacetylene (**2**), maltol glycoside (**5**), benzofuran derivative (**6**), and saponins (**7**–**11**) through their spectroscopic data compared to the literature data. Among the isolated compounds, compounds **2**–**5**, **7**, **9**, and **10** showed inhibitory effects on inflammatory cytokines (IL-1β, IL-6, and TNF-α) related to atopic dermatitis against TNF-α/IFN-γ-stimulated HaCaT keratinocytes at 10 μM. Notably, astersaponin J (**7**) exhibited a dose-dependent reduction in IL-1β, IL-6, and TNF-α levels, suggesting that it could be a potential candidate of a therapeutic agent for atopic dermatitis.

## Figures and Tables

**Table 1:** ^1^H and ^13^C NMR spectroscopic data of compounds **1** and **3** (*δ* in ppm; methanol-*d*_4_; 500 and 125 MHz).

Position *^a^*	1		3	
	*δ*_H_ Multi (*J* in Hz)	*δ*_C_	*δ*_H_ Multi (*J* in Hz)	*δ*_C_
1	3.97 dt (10.0, 5.5)/3.73 dt (10.0, 5.5)	67.1	-	177.4
2	1.97 q (6.0)	39.2	4.75 t (7.0)	61.1
3	4.65 t (7.0)	60.4	2.55 d (7.0)	45.6
4	-	83.9	-	83.5
5	-	69.9	-	69.6
6	-	72.6	-	72.7
7	-	78.4	-	78.2
8	5.59 dt (16.0, 2.0)	110.7	5.57 dd (16.0, 2.0)	110.7
9	6.33 dd (15.5, 7.0)	145.3	6.31 dq (16.0, 7.0)	145.2
10	1.81 dd (7.0, 2.0)	19.1	1.81 dd (7.0, 2.0)	19.0
11				
12				
13				
14				
15				
16				
17				
Glc-1′	4.26 d (8.0)	104.8		
Glc-2′	3.49 ddd (10.0, 8.5, 5.5)	73.7		
Glc-3′	3.43 ddd (8.0, 5.5, 3.0)	78.0		
Glc-4′	3.22 m	71.6		
Glc-5′	3.19 m	75.2		
Glc-6′	4.09 dd (11.0, 2.0)/3.87 dd (11.5, 5.5)	69.9		
Xyl-1″	4.31 d (7.5)	105.7		
Xyl-2″	3.74 m	77.1		
Xyl-3″	3.34 m	75.0		
Xyl-4″	3.87 m	71.3		
Xyl-5″	3.20 m	67.2		

***^a^*** All assignments were based on ^1^H-^1^H COSY, ^1^H-^13^C HSQC, and HMBC results.

**Table 2:** ^1^H and ^13^C NMR spectroscopic data of compound **4** (*δ* in ppm; methanol-*d*_4_; 500 and 125 MHz).

Position *^a^*	4	
	*δ*_H_ Multi (*J* in Hz)	*δ*_C_
1		128.4
2	7.72 d (1.5)	124.2
3		148.4
4		149.4
5	7.68 d (8.5)	124.0
6	7.81 dd (8.5, 1.5)	127.2
7		167.5
8		169.2
9		157.4
10	5.44 d (1.5)/4.43 d (1.5)	98.6
7-OCH_3_	3.88 s	52.7

***^a^*** All assignments were based on ^1^H-^1^H COSY, ^1^H-^13^C HSQC, and HMBC results.

## Data Availability

The data of this study are available from the corresponding author upon reasonable request.

## Supplementary Materials

The following supporting information can be downloaded at https://www.mdpi.com/article/10.3390/molecules29215002/s1, Figure S1: HR-ESI-MS spectrum of compound **1**; Figure S2: ^1^H NMR spectrum of compound **1** (500 MHz; methanol-*d*_4_); Figure S3: ^13^C NMR spectrum of compound **1** (125 MHz; methanol-*d*_4_); Figure S4: ^1^H-^13^C HSQC spectrum of compound **1**; Figure S5: ^1^H-^1^H COSY spectrum of compound **1**; Figure S6: ^1^H-^13^C HMBC spectrum of compound **1**; Figure S7: ^1^H-^1^H NOESY spectrum of compound **1**; Figure S8: HR-ESI-MS spectrum of compound **3**; Figure S9: ^1^H NMR spectrum of compound **3** (500 MHz; methanol-*d*_4_); Figure S10: ^13^C NMR spectrum of compound **3** (125 MHz; methanol-*d*_4_); Figure S11: ^1^H-^13^C HMBC spectrum of compound **3**; Figure S12: HR-ESI-MS spectrum of compound **4**; Figure S13: ^1^H NMR spectrum of compound **4** (500 MHz; methanol-*d*_4_); Figure S14: ^13^C NMR spectrum of compound **4** (125 MHz; methanol-*d*_4_); Figure S15: ^1^H-^13^C HSQC spectrum of compound **4**; Figure S16: ^1^H-^1^H COSY spectrum of compound **5**; Figure S17: ^1^H-^13^C HMBC spectrum of compound **4**; Figure S18: Acid hydrolysis result for compound **1**. A: Extracted ion chromatogram (EIC) of derivatized d-glucose and l-glucose (*m*/*z* = 444.5–445.5) B: MS/MS spectrum of d-glucose (left) and compound **1** (right; *t*_R_ 14.1 min); Figure S19: Acid hydrolysis result for compound **1**. A: Extracted ion chromatogram (EIC) of derivatized d-xylose and l-xylose (*m*/*z* = 414.5–415.5) B: MS/MS spectrum of d-xylose (left) and compound **1** (right; *t*_R_ 14.6 min).
