# Supplementary material for: Discovery of Chemical Constituents with Anti-Atopic Dermatitis Properties from Aster koraiensis
Source: Molecules. 2024 Oct 22;29(21):5002. doi: 10.3390/molecules29215002 (PMC11547852; doi:10.3390/molecules29215002)

Supplementary material for

Discovery of chemical constituents with anti-atopic dermatitis properties from *Aster koraiensis*

List of Contents

Figure S1. HR-ESI-MS spectrum of compound **1**

Figure S2. ^{1}H NMR spectrum of compound **1** (500 MHz, methanol-d_4)

Figure S3. ^{13}C NMR spectrum of compound **1** (125 MHz, methanol-d_4)

Figure S4. ^{1}H-^{13}C HSQC spectrum of compound **1**

Figure S5. ^{1}H-^{1}H COSY spectrum of compound **1**

Figure S6. ^{1}H-^{13}C HMBC spectrum of compound **1**

Figure S7. ^{1}H-^{1}H NOESY spectrum of compound **1**

Figure S8. HR-ESI-MS spectrum of compound **3**

Figure S9. ^{1}H NMR spectrum of compound **3** (500 MHz, methanol-d_4)

Figure S10. ^{13}C NMR spectrum of compound **3** (125 MHz, methanol-d_4)

Figure S11. ^{1}H-^{13}C HMBC spectrum of compound **3**

Figure S12. HR-ESI-MS spectrum of compound **4**

Figure S13. ^{1}H NMR spectrum of compound **4** (500 MHz, methanol-d_4)

Figure S14. ^{13}C NMR spectrum of compound **4** (125 MHz, methanol-d_4)

Figure S15. ^{1}H-^{13}C HSQC spectrum of compound **4**

Figure S16. ^{1}H-^{1}H COSY spectrum of compound **4**

Figure S17. ^{1}H-^{13}C HMBC spectrum of compound **4**

Figure S18. Acid hydrolysis result for compound **1**. A: Extracted ion chromatogram (EIC) of derivatized D-glucose and L-glucose (m/z = 444.5–445.5) B: MS/MS spectrum of d-glucose (left) and compound **1** (right; t_R 14.1 min)

Figure S19. Acid hydrolysis result for compound **1**. A: Extracted ion chromatogram (EIC) of derivatized D-xylose and L-xylose (m/z = 414.5–415.5) B: MS/MS spectrum of D-xylose (left) and compound **1** (right; t_R 14.6 min)

Figure S1. HR-ESI-MS spectrum of compound **1**

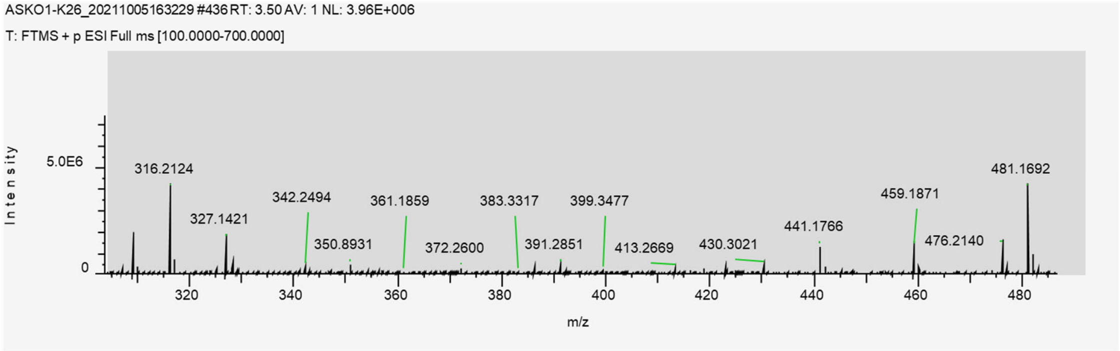

Figure S2. ^{1}H NMR spectrum of compound **1** (500 MHz, methanol-d_4)

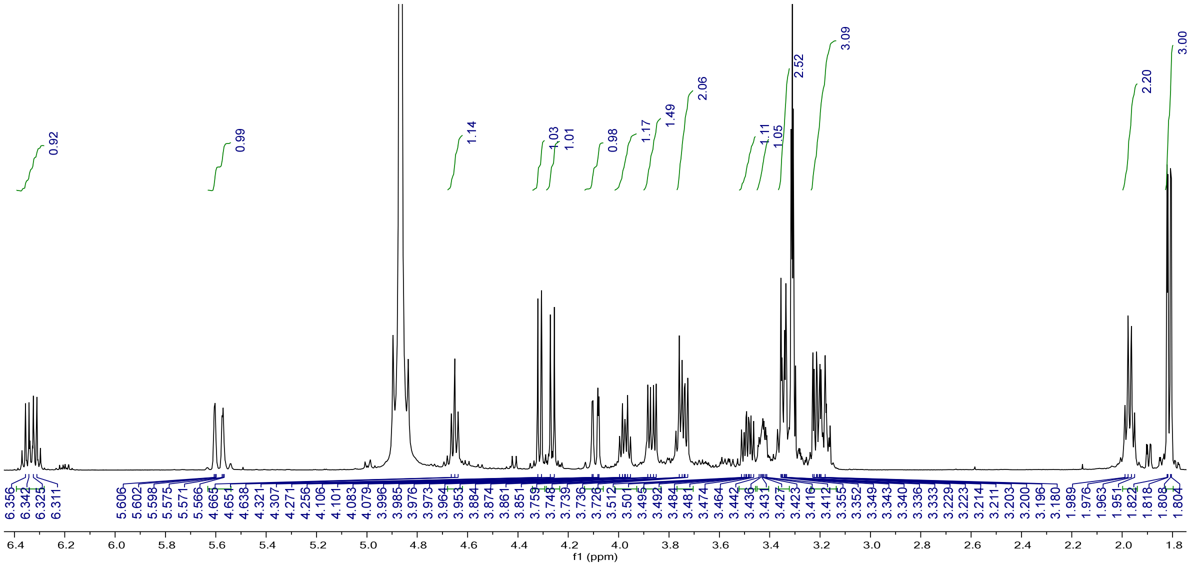

Figure S3. ^{13}C NMR spectrum of compound **1** (125 MHz, methanol-d_4)

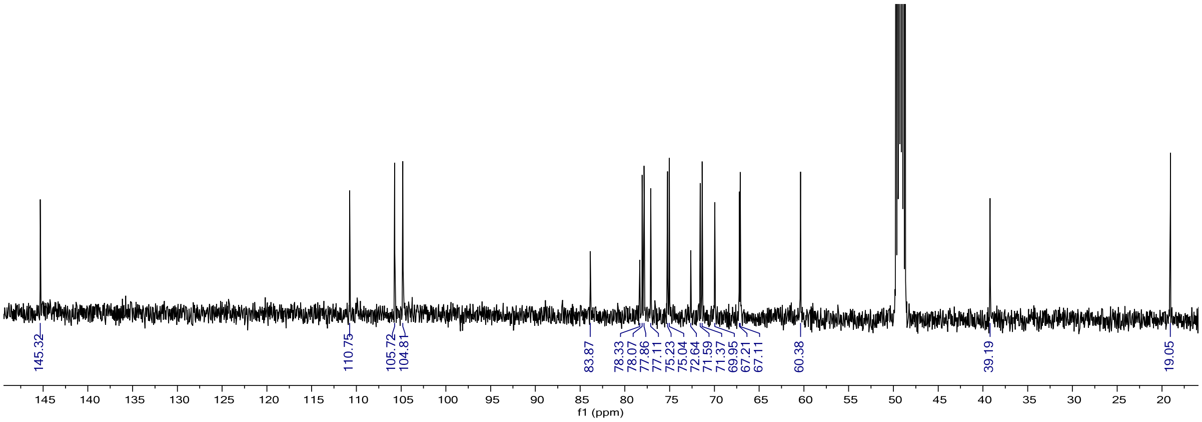

Figure S4. ^{1}H-^{13}C HSQC spectrum of compound **1**

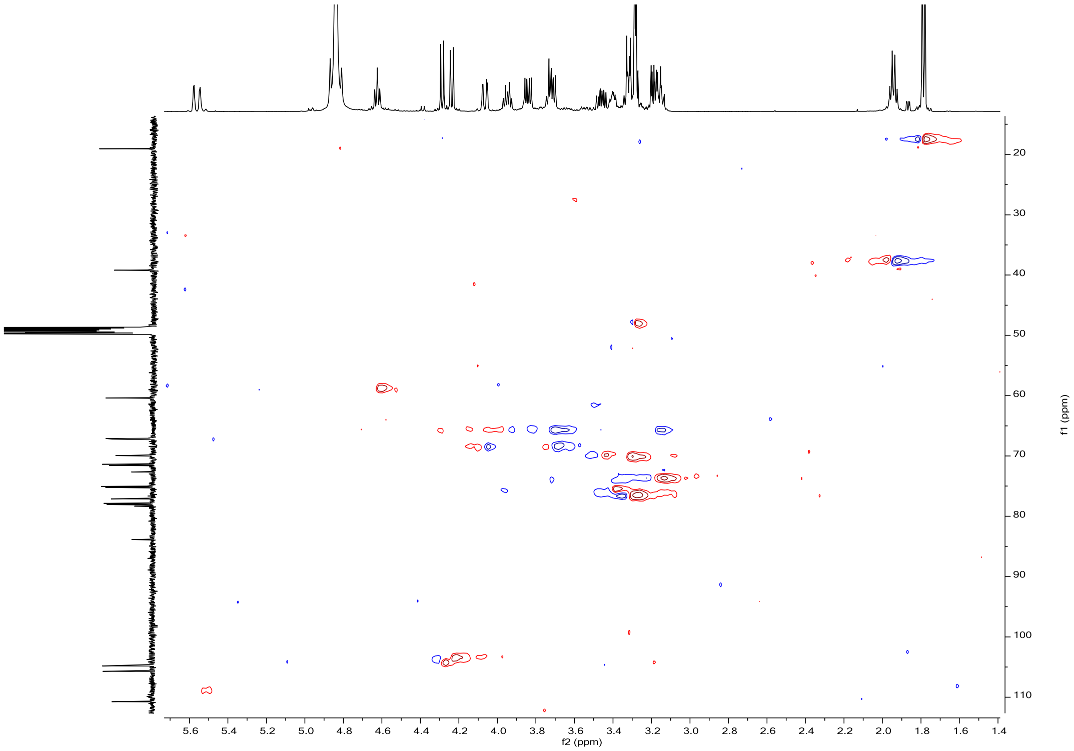

Figure S5. ^{1}H-^{1}H COSY spectrum of compound **1**

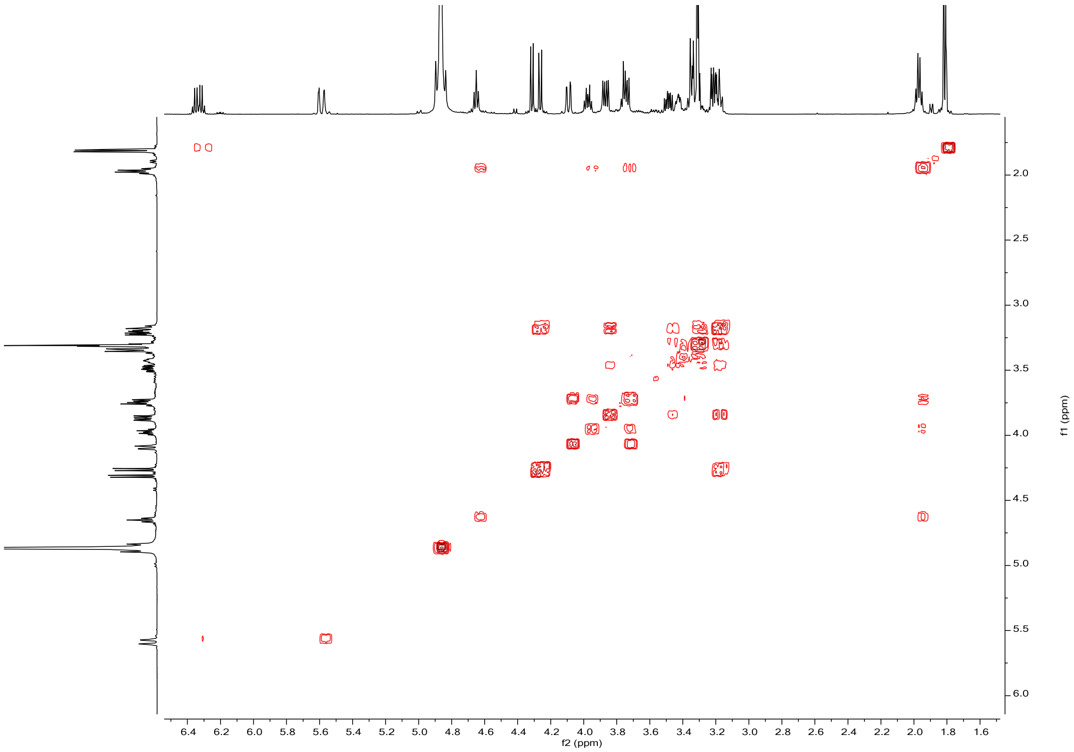

Figure S6. ^{1}H-^{13}C HMBC spectrum of compound **1**

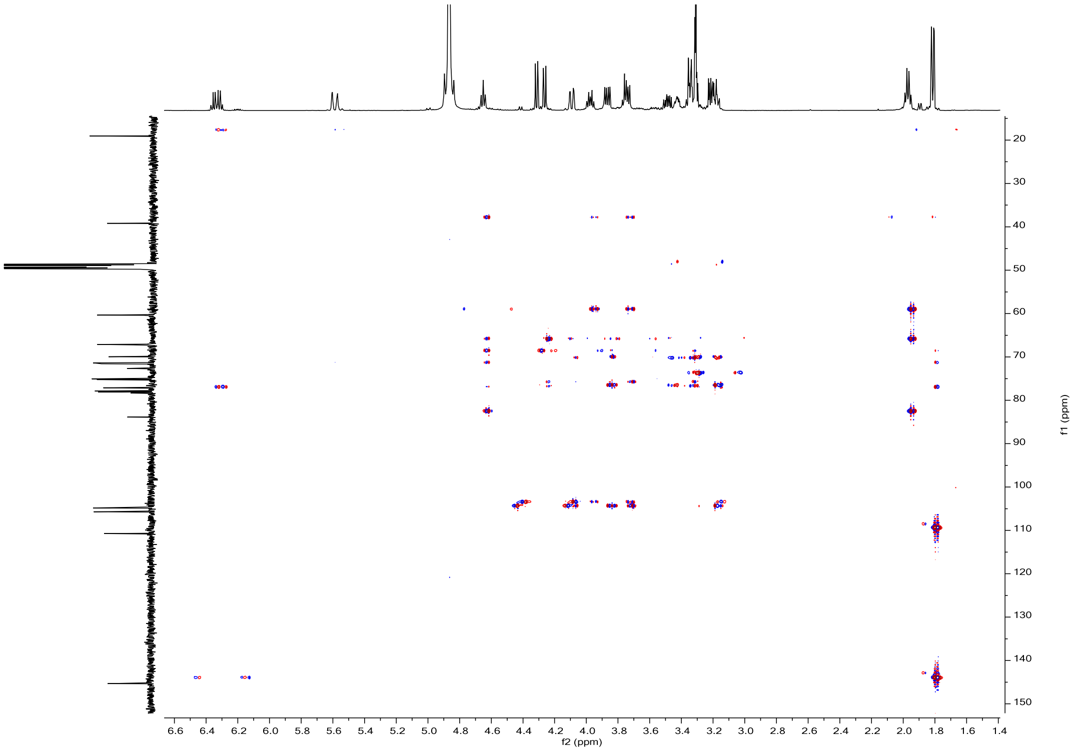

Figure S7. ^{1}H-^{1}H NOESY spectrum of compound **1**

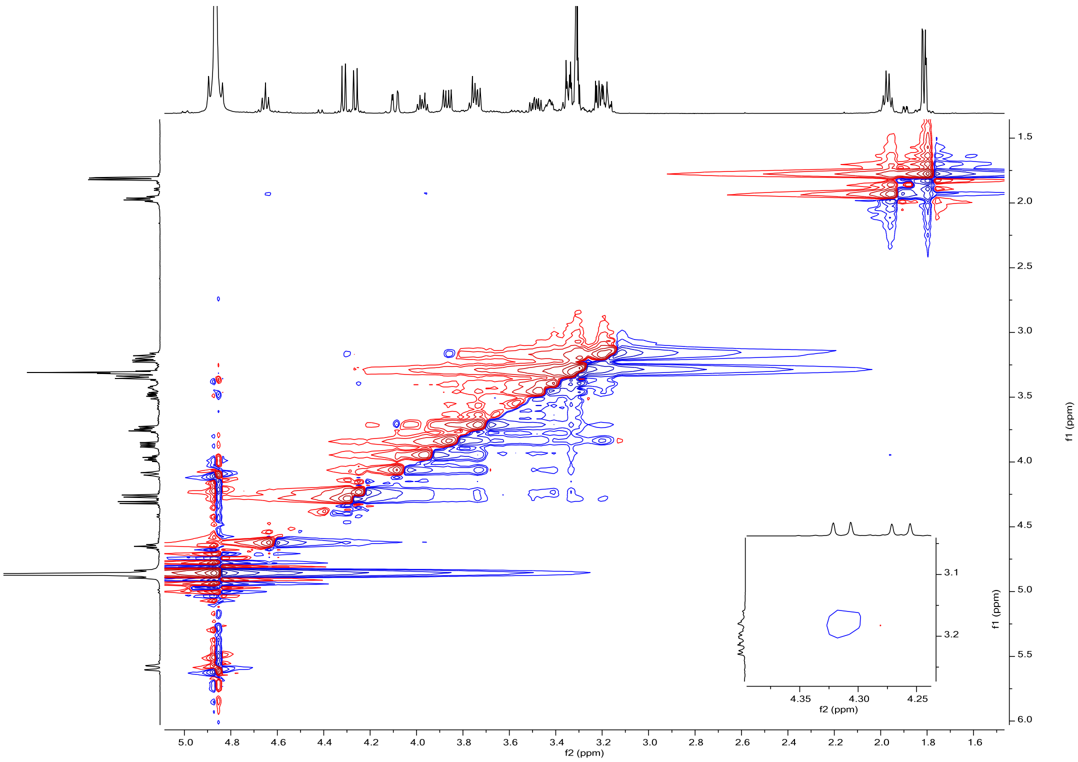

Figure S8. HR-ESI-MS spectrum of compound **3**

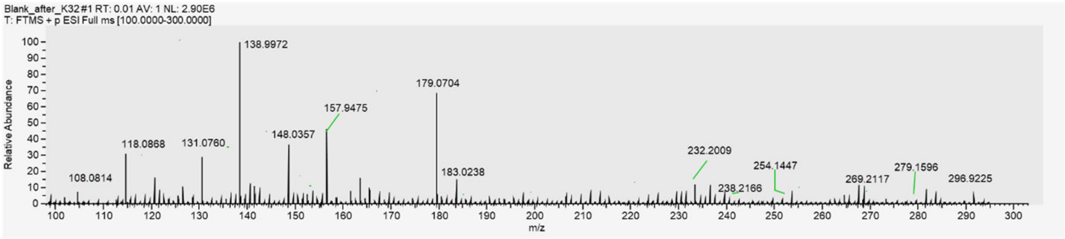

Figure S9. ^{1}H NMR spectrum of compound **3** (500 MHz, methanol-d_4)

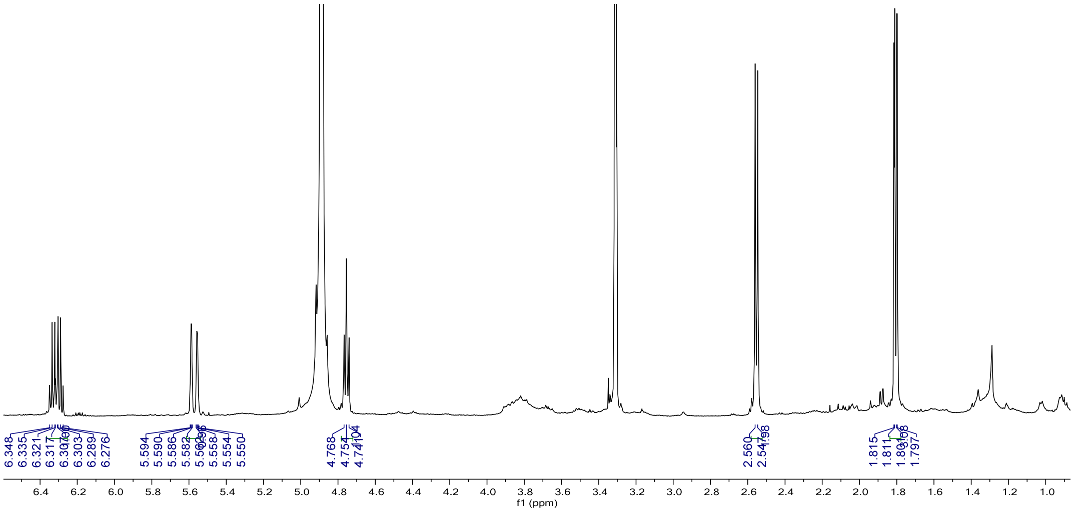

Figure S10. ^{13}C NMR spectrum of compound **3** (125 MHz, methanol-d_4)

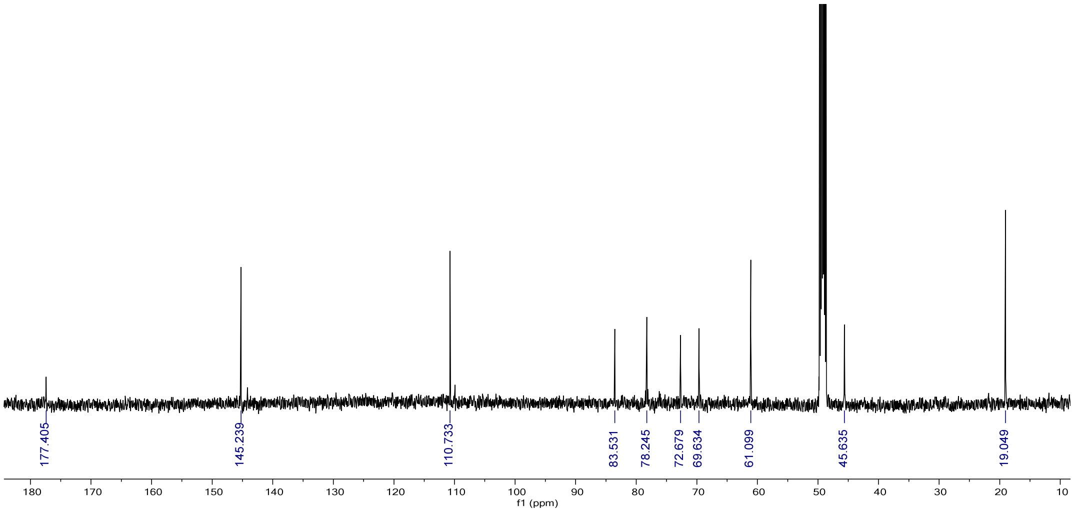

Figure S11. ^{1}H-^{13}C HMBC spectrum of compound **3**

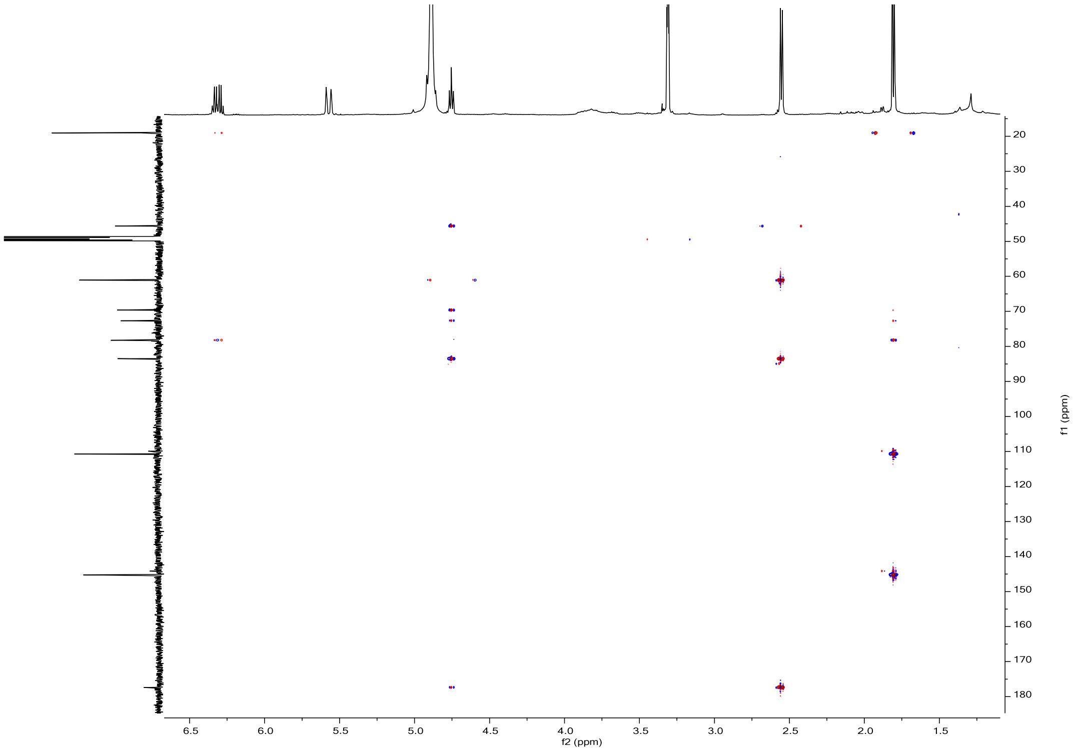

Figure S12. HR-ESI-MS spectrum of compound **4**

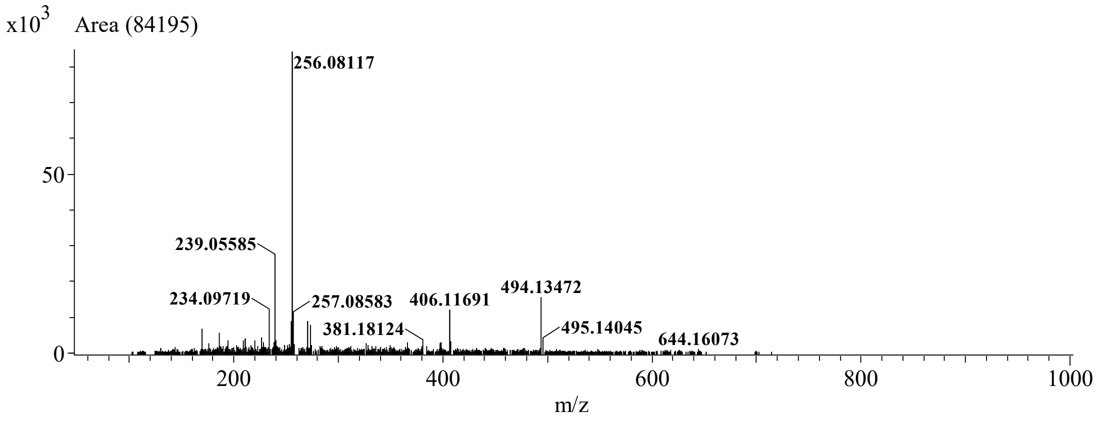

Figure S13. ^{1}H NMR spectrum of compound **4** (500 MHz, methanol-d_4)

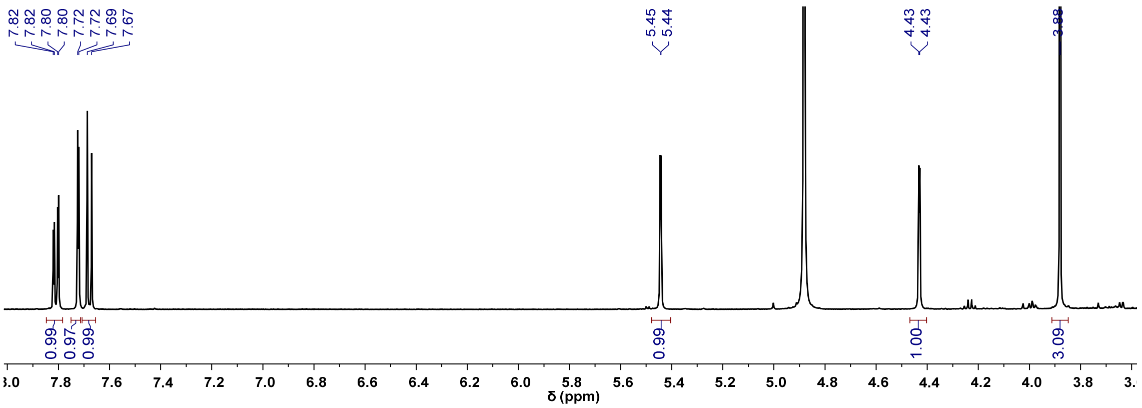

Figure S14. ^{13}C NMR spectrum of compound **4** (125 MHz, methanol-d_4)

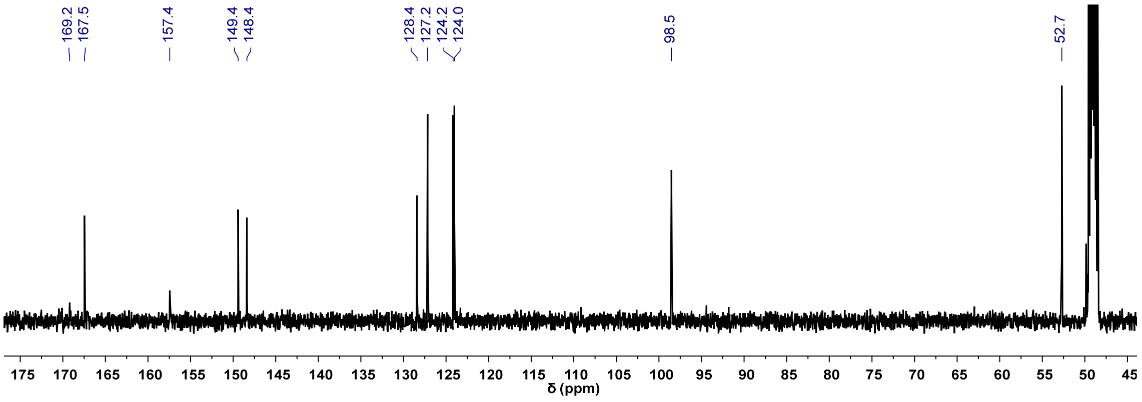

Figure S15. ^{1}H-^{13}C HSQC spectrum of compound **4**

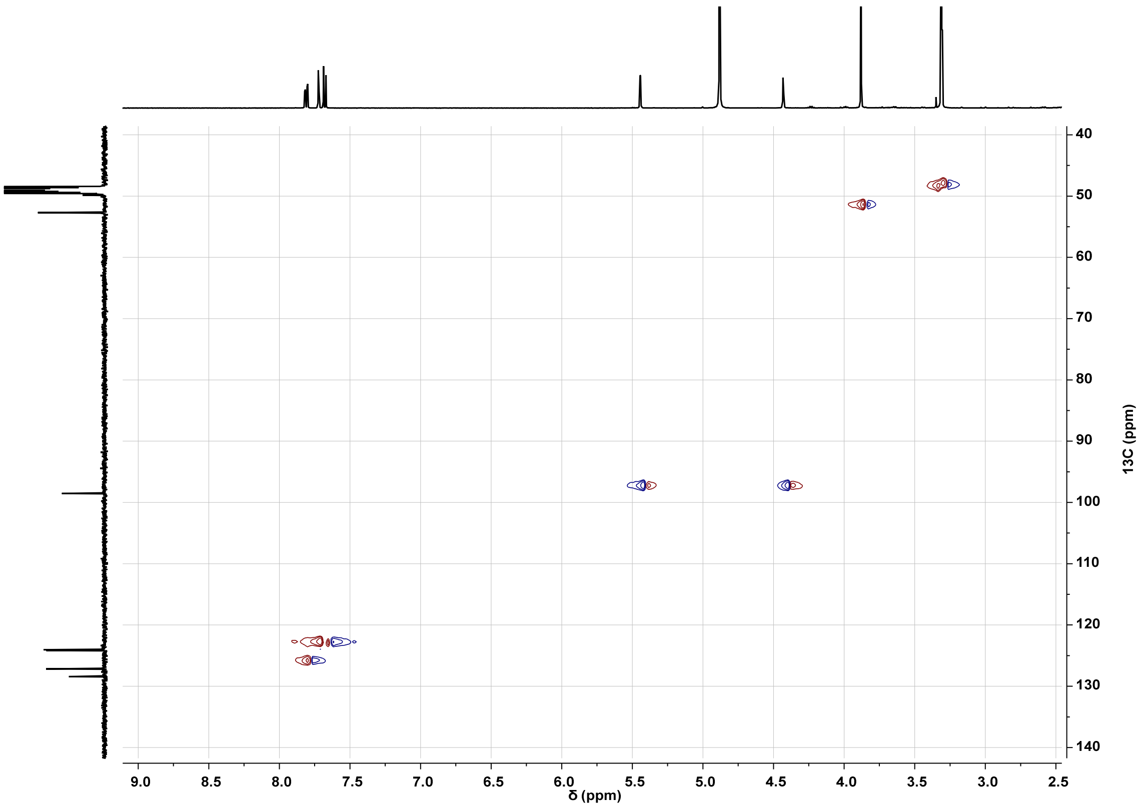

Figure S16. ^{1}H-^{1}H COSY spectrum of compound **4**

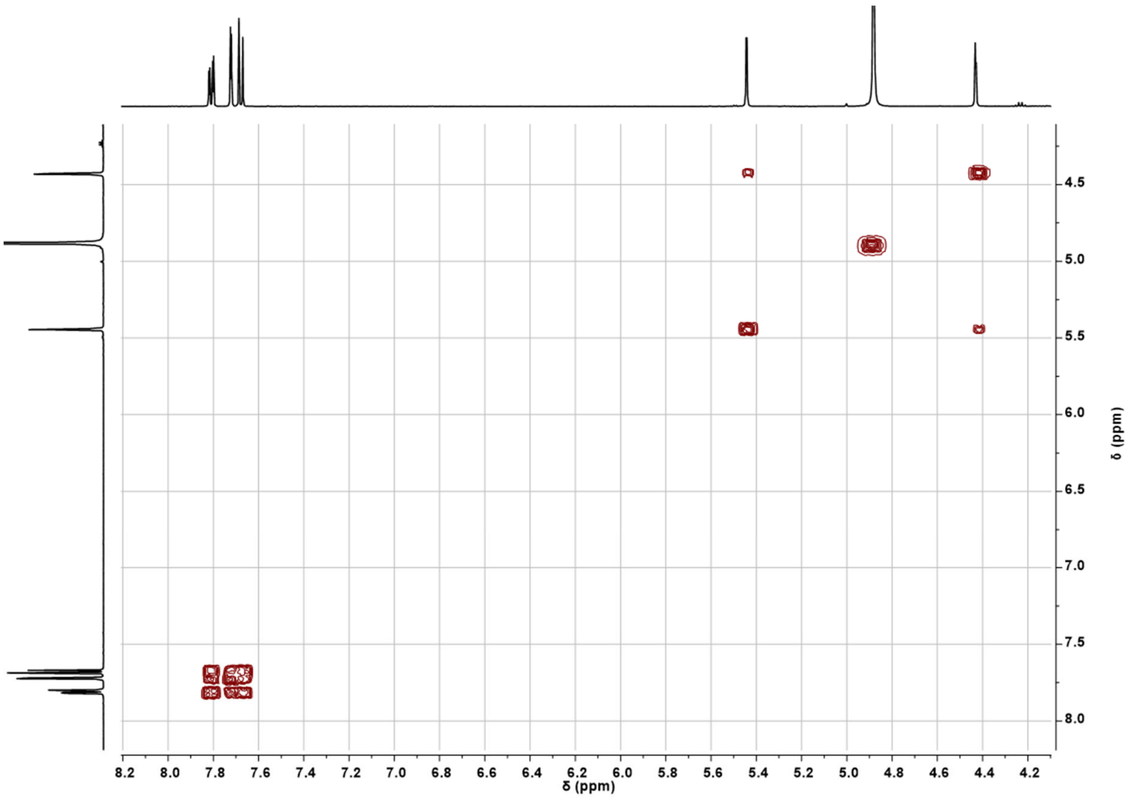

Figure S17. ^{1}H-^{13}C HMBC spectrum of compound **4**

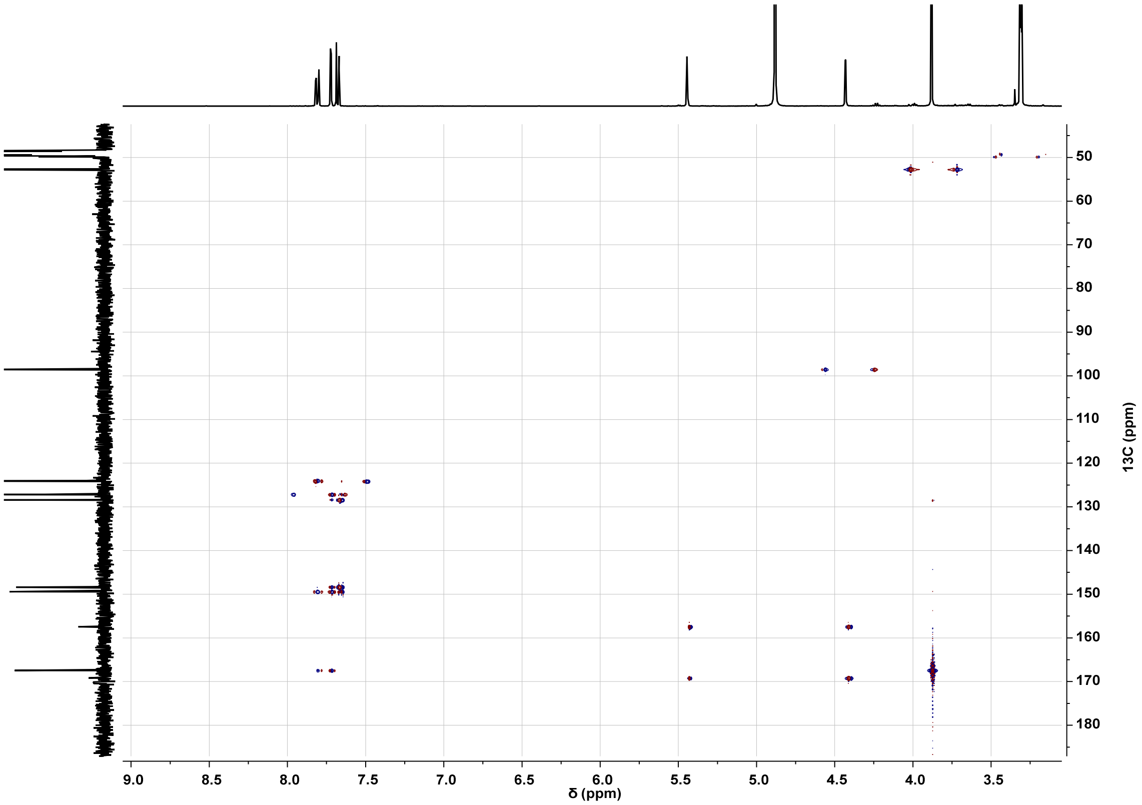

Figure S18. Acid hydrolysis result for compound **1**. A: Extracted ion chromatogram (EIC) of derivatized D-glucose and L-glucose (*m/z* = 444.5–445.5) B: MS/MS spectrum of d-glucose (left) and compound **1** (right; t_R 14.1 min)

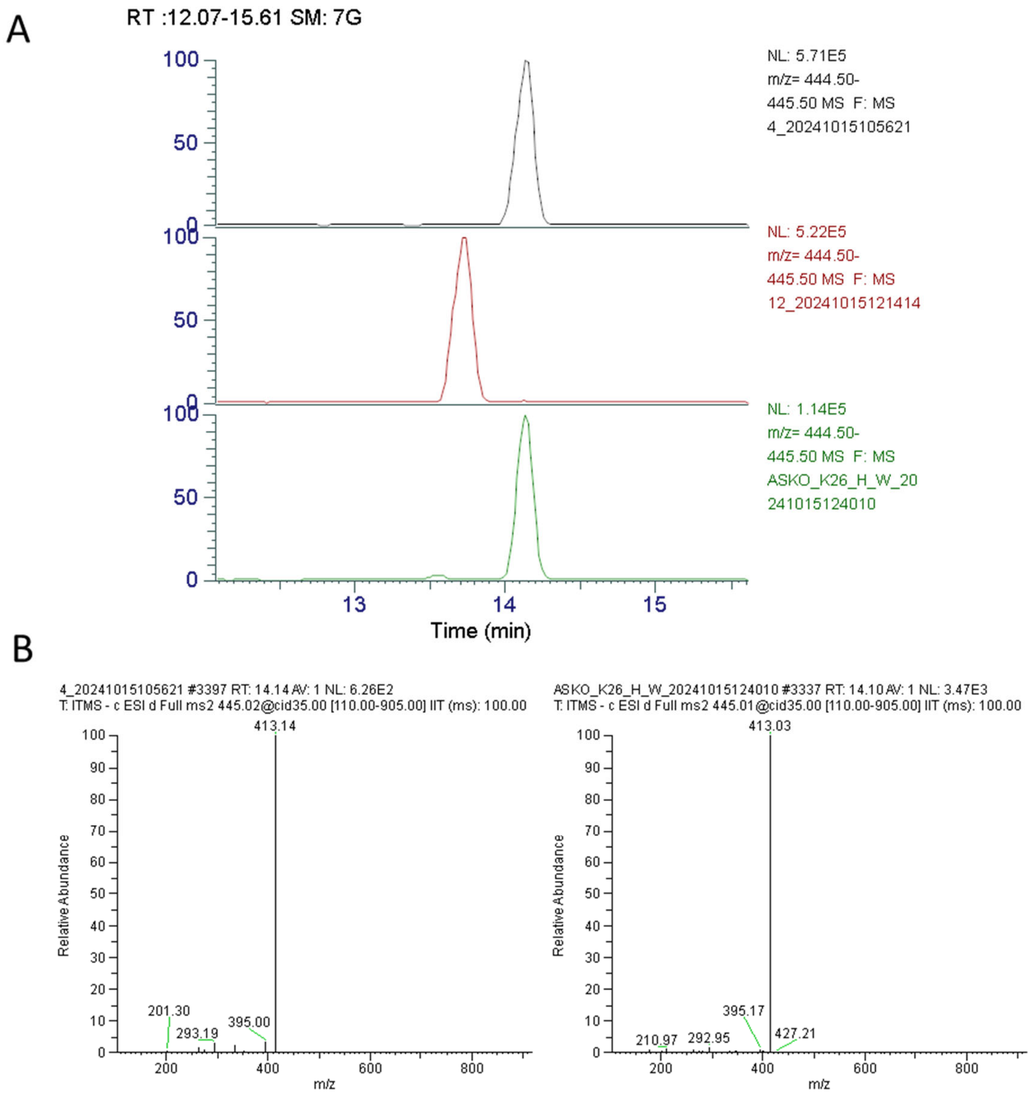

Figure S19. Acid hydrolysis result for compound **1**. A: Extracted ion chromatogram (EIC) of derivatized D-xylose and L-xylose (m/z = 414.5–415.5) B: MS/MS spectrum of D-xylose (left) and compound **1** (right; t_R 14.6 min)

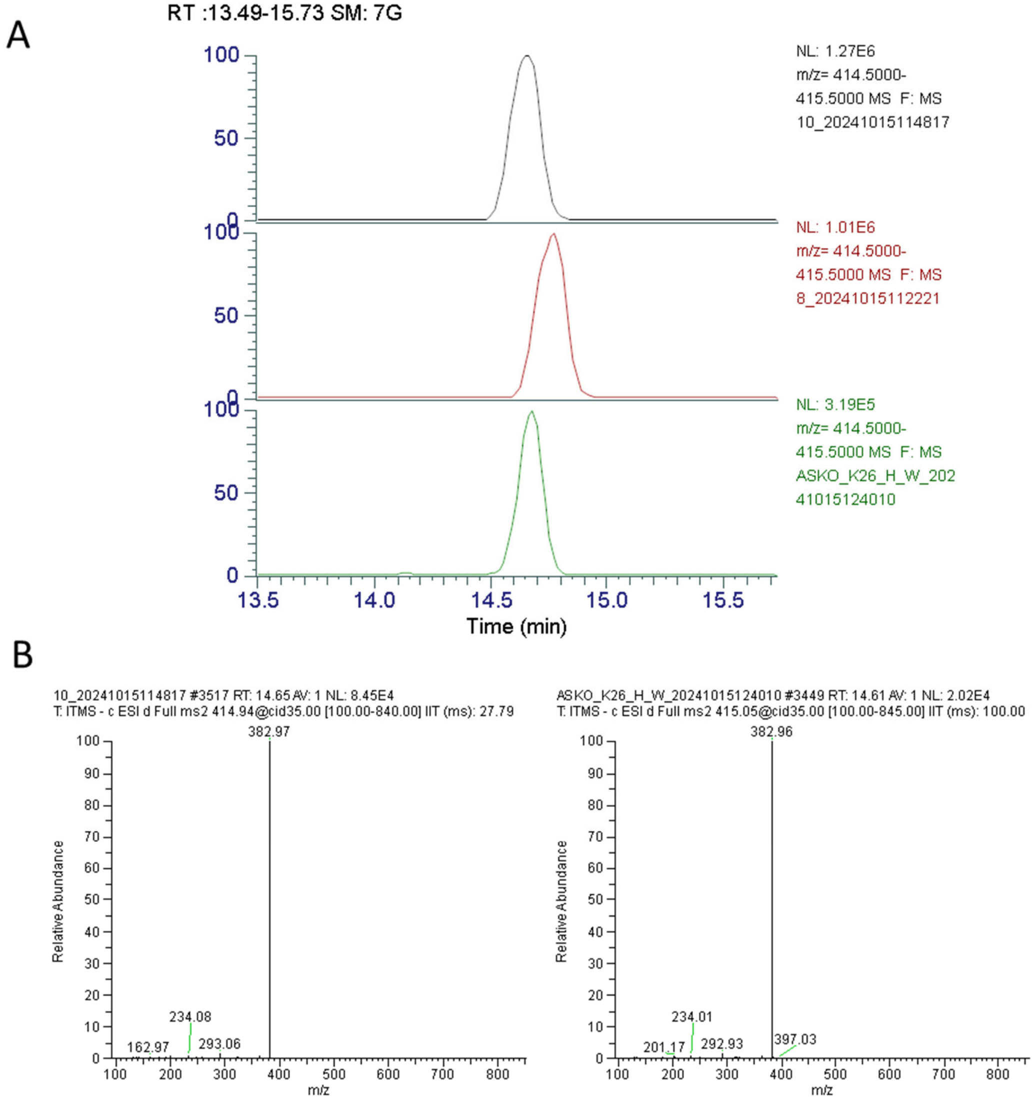

Supplement: Supplementary file 1 [file molecules-29-05002-s001.zip › molecules-3226442-supplementary.pdf]
